# Magnitude of the Cytomegalovirus infection among pregnant women attending antenatal clinics in the city of Mwanza, Tanzania

**DOI:** 10.1186/s13104-017-2813-4

**Published:** 2017-09-20

**Authors:** Elieza Chibwe, Mariam M. Mirambo, Albert Kihunrwa, Stephen E. Mshana

**Affiliations:** 1 Department of Obstetrics and Gynecology, Weill Bugando School of Medicine, P.O. Box 1464, Mwanza, Tanzania; 2 Department of Microbiology and Immunology, Weill Bugando School of Medicine, P.O. Box 1464, Mwanza, Tanzania

**Keywords:** Cytomegalovirus, Low socioeconomic status, Pregnant women

## Abstract

**Background:**

Despite, Cytomegalovirus (CMV) infection being associated with a potential risk to the fetus, there is limited data from Tanzania and many other developing countries regarding the epidemiology and the impact of CMV infections. This cross-sectional study was conducted between December 2014 and June 2015 among pregnant women attending antenatal clinics in the city of Mwanza, Tanzania to investigate the magnitude and associated factors of CMV infection.

**Methods:**

The specific CMV IgM and IgG antibodies were detected using indirect enzyme linked immunosorbent assay (ELISA). Demographic and clinical data were collected using pre-tested data collection tool. Data were analysed using STATA version 13.

**Results:**

A total of 261 pregnant women with median age of 20 (IQR 19–25) years and mean gestation age of 17 ± 7.8 weeks were enrolled. The seroprevalence of CMV IgG antibodies was 193(73.9%; 95% CI 67.2–79.6) while that of CMV IgM antibodies was 0.4%. As the age increased by one unit the IgG seroprevalence was found to increase by 0.3% (95% CI 0.13–0.47, p = 0.001) whereas the risk of being IgG positive increased by 24%. On multivariable logistic regression analysis only urban residence (OR 6.329, 95% CI 2.885–13.887, p < 0.001) was found to independently predict CMV IgG seropositivity. Regarding the outcomes of previous pregnancies the history of miscarriage independently predicted IgG seropositivity (OR 5.6, 95% CI 1.29–24.178, p = 0.021). The IgM seropositive woman had fatal outcome of the term delivery of the baby with microcephaly and spinal-bifida.

**Conclusion:**

Cytomegalovirus seroprevalence among pregnant women residing in urban areas of Mwanza city, Tanzania is high and is associated with poor pregnancy outcomes. There is a need to emphasize routine screening of CMV in order to establish the impact of CMV during pregnancy.

## Background

Primary Cytomegalovirus (CMV) infection during pregnancy poses a serious threat to the fetus and newborn. CMV is ubiquitous in nature and can be transmitted through direct contact or vertically from pregnant women to the fetus [1]. In contrast to other congenital infections such as rubella virus [2], CMV infection can be primary, re-infection or reactivation [3]. Primary maternal infection is associated with 30–40% risk of fetal infections, and 20–25% risk of adverse pregnancy outcomes [4]. Intrauterine infections can cause fetal death in approximately 10% of fetuses [5]. Approximately 15% of newborns with congenital infections develop neurologic disorders such as sensory-neural hearing loss, mental retardation and delayed milestone [6].

The seroprevalence of CMV among pregnant women has been found to be high [7]; with highest seroprevalence being reported in Turkey, Qatar, Saudi Arabia, Taiwan and sub-Saharan Africa [8–16]. In Tanzania, there is limited information about the magnitude of CMV among pregnant women with no single report from Mwanza city despite having high prevalence of the congenital defects with unidentified causes [17, 18]. Considering its potential in causing congenital defects; it is important to understand the magnitude and associated factors of CMV among pregnant women. Therefore, this study for the first time in Tanzania has established that there is high seroprevalence of CMV infection among pregnant women and is associated with adverse pregnancy outcomes; the information that may be important to reinforce its control measures in the developing countries.

## Methods

A cross sectional hospital based study was conducted from December 2014 to July 2015 among pregnant women attending Karume (Rural) and Makongoro (Urban) antenatal clinics. About 4–5 ml of blood was collected using plain vacutainer tubes (Becton, Dickinson and Company, Nairobi, Kenya) and transported to the Bugando Medical Centre laboratory. Sera were separated and stored at −80 °C until processing.

Socio-demographic data were collected using pre-tested questionnaire. Data collected included; age, parity, gestation age, the outcome of the previous pregnancies, education level, residence, and socio-economic status. Good social economic status was defined as being educated with sustainable income or having reliable income generating activities [19].

### Laboratory procedures

Detection of the specific Cytomegalovirus (CMV) IgM and IgG antibodies were done using commercial indirect enzyme-linked immunosorbent assay(PishtazTeb, Tehran, Iran) according to the manufacturer’s instructions using automated ELISA system (ChemWell^®^ 2910-Awareness Technology Inc. USA). The ELISA assays have been found to have sensitivity and specificity of > 95% [20–23]. The system was calibrated using provided standards and controls before any sample testing was done. In addition HIV testing was done to all samples based on the national rapid test algorithm [24].

### Data management and analysis

Data were entered in the computer using Microsoft Office Excel 2007 and analyzed using the STATA version 11 (College Station, Texas, USA). Continuous variables were summarized as median with interquartile range while categorical variables were summarized as proportions. Univariable and multivariable logistic regression analysis was done to investigate factors associated with CMV seropositivity. For factors associated with IgG seropositivity, all factors found to be significant on univariable analysis were subjected to the multivariable logistic regression analysis while for the relation between the outcome of the previous pregnancies and CMV IgG seropositivity all factors were fitted in the multivariable model. Odds ratio (OR) and 95% confidence interval were noted and a p value of < 0.05 was considered significant.

## Results

### Demographic characteristics

The median age of the 261 enrolled women was 20 (IQR 19–25) years with mean gestation age of 17 ± 7.8 weeks. One hundred and five (40.2%), 115 (44.06%) and 41 (15.7%) were in the first, second and third trimester respectively. As defined by the Mwanza municipal city council; the majority of women 169 (64.8%) were from urban areas. Also it was observed that the majority of women 192 (73.9%) had poor socioeconomic status. Out of 261 pregnant women, 22 (8.4%) were found to be HIV positive.

### Seroprevalence of CMV and associated factors

**Fig. 1:**
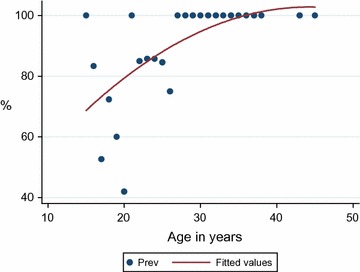
A correlation between CMV sero-prevalence and maternal age

Overall seroprevalence of CMV IgM specific antibodies was 1 (0.4%) while for IgG specific antibodies the seroprevalence was 193 (73.9%, 95% CI 68.5–79.2). On Wilcoxon Ranksum–Mann–Whitney test; the median age of IgG seropositive women was significantly high compared to that of IgG seronegative women [22 (IQR 19–27) vs. 20 (IQR 18–20), p < 0.001]. As the age increases by one year the seroprevalence was found to increase by 0.3% (95% CI 0.13–0.47, p = 0.001) while the risk of being CMV IgG positive increased by 24% (Fig. 1). On univariable logistic regression analysis, the IgG seropositivity was found to decrease significantly as gestation age increases (OR 0.1, 95% CI 1.04–2.23, p < 0.001). The mean gestation age of IgG seropositive women was significantly lower than the mean gestation age of IgG seronegative women (15.1 ± 6.8 vs. 22 ± 7.9, p = 0.001).

Increased in age, being multiparous and low socioeconomic status were significantly found to be associated with CMV IgG seroprevalence (p < 0.01) Table 1. On multivariable logistic regression analysis only urban residence was found to be an independent predictor of CMV IgG seropositivity among pregnant women (OR: 6.34; 95% CI 2.9–13.9, p < 0.001).

**Table 1:** Univariable and multivariable logistic regression analysis on factors associated with CMV IgG seropositivity among women attending antenatal clinics in Mwanza city

Characteristics	IgG seropositivity (N %)	Univariable: OR (95% CI)	*p* value	Multivariable OR (95% CI)	p value
Age	20 (IQR: 19–25)	1.2 (1.133–1.368)	< 0.001	1.08 (0.97–1.20)	0.175
Gestation age (wks)	22.6 ± 7.9	0.880 (0.846-0.916)	< 0.001	0.970 (0.923–1.019)	0.220
Parity					
Multipara (144)	94 (65.28)	1			
Nullipara 117	99 (84.62)	2.92 (1.59–5.37)	< 0.001	1.43 (0.63–3.22)	0.388
Residence					
Rural (92)	40 (43.48)	1			
Urban (169)	153 (90.53)	12.43 (6.43–24.04)	< 0.001	6.34 (2.89–13.89)	< 0.001
SES					
High (69)	128 (66.67)	1			
Low (192)	65 (94.20)	8.12 (2.83–23.30)	< 0.001	2.847 (0.89–9.15)	0.079
HIV Status					
Negative	174 (72.8)	1			
Positive	19 (86.4)	2.36 (0.67–8.26)	0.177		
History of BT					
Yes (8)	6 (75)	1			
No (253)	187 (73.95)	1.056 (0.21–5.38)	0.945		
Nursery school baby					
Yes (7)	6 (85.714)	1			
No (84)	34 (40.48%)	2.150 (0.254–18.186)	0.482		

*SES* Socio-economic status

### Outcome of previous pregnancies and current pregnancy for IgM positive case

Based on the outcomes of the previous pregnancies; all women with previous history of still birth and history of giving birth to a baby with congenital malformations were CMV IgG sero-positive. In addition, women with history of miscarriage had significantly high IgG seroprevalence than those with no history of miscarriage (93.3% vs. 71%, p = 0.021) Table 2. The history of miscarriage remained to be significant factor associated with IgG seropositivity on the multivariable logistic regression analysis. IgM positive participant had term delivery of a baby with microcephaly and spinal-bifida; the baby died within 2 h of delivery.

**Table 2:** Outcome of previous pregnancy in relation to CMV IgG seropositivity among women attending antenatal clinics in the city of Mwanza, Tanzania

Characteristics	IgG seropositivity (N, %)	Univariate		Multivariate	
		OR (95%CI)	p value	OR (95%CI)	p value
Previous history of miscarriage					
No (231)	165 (71.43)	1			
Yes (30)	28 (93.33)	5.6 (1.29–24.178)	0.021	5.2 (1.1–24.40)	0.038
Previous baby with LBW					
No (237)	175 (73.84)	1			
Yes (24)	18 (75)	1.062 (0.404–2.799)	0.902	0.5 (0.15–1.68)	0.267
Previous stillbirth					
No 257	189 (73.54)				
Yes (4)	4 (100.00)	Omitted			
Previous preterm					
No (248)	182(73.39)	1			
Yes (13)	11 (84.62)	1.994 (0.431–9.236)	0.377	1.3 (0.2–8.40)	0.780
Previous baby with malformation					
No (255)	187 (73.33)				
Yes (6)	6 (100.00)	Omitted			

*LBW* Low birth weight, *Omitted* Perfect predictor

## Discussion

Understanding the magnitude of the Cytomegalovirus (CMV) infections among pregnant women is essential for exploring its epidemiology and control measures. The CMV has been found to be among the common cause of hearing loss and mental retardation among many other defects in congenitally infected children [25–27]. However; the epidemiology of CMV infections and its impact is limited in the many developing countries including Tanzania. As observed in previous studies in developing countries [8, 12, 13, 16], a significant proportion of pregnant women were found to be CMV IgG seropositive. The results from this study and other studies in the developing countries call for a joint effort to control CMV infections considering its potential effect in causing congenital defects and poor pregnancy outcomes. The CMV management guidelines provide a framework for the diagnosis and management of suspected CMV infections with the emphasis on the importance of screening and follow up using ultrasound [4]. Women with primary infections during pregnancy should be informed about a 30–40% risk of the intrauterine transmission and fetal infections, and a risk of 20–25% of developing sequelae if the fetus is infected. The use of passive immunization (CMV-hyper-immune globulin) has been found to be beneficial in preventing congenital anomalies [28].

As per previous observations in other studies [13, 29], the acute CMV infection as indicated by positive CMV IgM antibodies was found to be very low in the current study. This could be explained by the fact that the accuracy of IgM antibodies in predicting primary infection is not reliable as the IgM may persist for several months after primary infection as well as in cases of reactivation or re-infection [30]. In most cases there is a need to perform IgG avidity test as an alternative to provide the status of active infection [31–33]. Due to the limited resources this this could not be done in our study.

Despite the fact that the trend on reactivation and re-infection were not established in this study; the high IgG seropositivity is alarming which calls for the need to screen these women for active infections for the proper management provision. The importance of these results is further supported by the fact that all women with history of stillbirth and child with congenital malformations were IgG seropositive. In addition, the IgM seropositive woman in the present study had poor pregnancy outcome. Further studies on the impact of CMV on poor pregnancy outcomes are highly recommended in the developing countries.

Residing in urban areas independently predicted CMV IgG seropositivity which is inconsistent to what was reported earlier [34]. The majority of population in the city of Mwanza reside in highly populated squatters with close contacts which favors transmission of airborne diseases. Also, on univariable analysis, increase in age was significantly found to be associated with IgG seropositivity, this confirms what was observed in the previous studies [3, 34–39]. Being the childhood illness and endemic in most of the sub-Saharan African countries; there is possibility that most of these women were either exposed during childhood and as the age increases are more likely to be infected. Though borderline significant in multivariable analysis, low socioeconomic status was found to predict CMV IgG seropositivity which is similar to what was observed in the previous studies [34, 40]. This could be explained by poor hygienic conditions which have been found to perpetuate the cycle of CMV transmission in the developing countries [41]. The decrease in CMV IgG seropositivity with gestation age observed in the current study might be due to pregnancy hemodilution [42].

HIV infected women were more likely to be CMV IgG seropositive than HIV negative; however the difference was not statistically significant. Of note was that a total of 57.8% of the pregnant women had no HIV results by the time of the recruitment which is against the WHO recommendations. WHO recommends that all the pregnant women should be counseled and tested for HIV at the first antenatal visit. There is a need to improve antenatal services in our setting to ensure all women are counseled and tested for HIV.

In this study one of the limitations is failure to detect CMV DNA that indicates active shedding of CMV which is an important risk factor for vertical transmission.

## Conclusion

Cytomegalovirus seroprevalence among pregnant women residing in urban areas of Mwanza city is significantly high and might be causing poor pregnancy outcomes such as miscarriage, congenital anomalies and stillbirth. This calls for the need to screen CMV infection during antenatal visits in order to provide appropriate management for the purpose of minimizing associated adverse outcomes. Further studies to investigate the outcome of pregnancy in relation to acute CMV infection are highly needed in the developing countries.

## Abbreviations

CMV: Cytomegalovirus
ELISA: enzyme linked immunosorbent assay
IQR: interquartile range
OR: odds ratio
